# Supplementary material for: Effectiveness of Somatosensory Stimulation for the Lower Limb and Foot to Improve Balance and Gait after Stroke: A Systematic Review
Source: Brain Sci. 2022 Aug 19;12(8):1102. doi: 10.3390/brainsci12081102 (PMC9405913; doi:10.3390/brainsci12081102)
Supplement: Supplementary file 1 [file brainsci-12-01102-s001.zip › brainsci-1853827-supplementary.pdf]

Table S1. Searches in the individual databases

Ageline

(((DE "Stroke") OR (DE "Brain Damage") OR (Stroke OR cerebrovascular accident OR cva OR acquired brain injury OR traumatic brain injury OR head injury OR tbi OR abi OR hemiplegia OR hemiparesis OR upper motor neuron lesion)) AND ((sens* OR stimulat* OR somatosens*) OR (propriocept* OR afferent OR (mobilisation OR mobilization OR mobilize OR mobilise) OR manipulat*)) AND ((DE "Feet") OR (DE "Legs") OR (foot OR leg OR lower limb OR lower extremity)) AND ((DE "Walking") OR (walk* OR gait OR mobil* OR step OR stance OR ambulat* OR weight bearing OR weight-bearing))) NOT (functional electrical stimulation OR FES)

AMED (Allied and Complementary Medicine)

1 stroke/
2 Cerebrovascular accident/
3 Brain injuries/
4 Head injuries/
5 Hemiplegia/
6 (stroke or cerebrovascular accident or CVA or acquired brain injury or traumatic brain injury or head injury or TBI or ABI or hemiplegia or hemiparesis or upper motor neuron lesion).mp. [mp=abstract, heading words, title]
7 1 or 2 or 3 or 4 or 5 or 6
8 Sensation disorders/
9 Electric stimulation/
10 Proprioception/
11 Mobilisation/
12 (sens* or stimulat* or somatosens* or propriocept* or afferent or mobilisation or manipulat* or sensory or sensation or sensibility).mp. [mp=abstract, heading words, title]
13 8 or 9 or 10 or 11 or 12
14 Foot/
15 Leg/
16 (foot or leg or lower limb or lower extremity).mp. [mp=abstract, heading words, title]
17 14 or 15 or 16
18 Walking/
19 Gait/
20 Weight bearing/
21 (walk* or gait or mobil* or step or stance or ambulat* or weight bearing or weight-bearing).mp. [mp=abstract, heading words, title]
22 18 or 19 or 20 or 21
23 randomised controlled trial.mp. [mp=abstract, heading words, title]
24 Randomized controlled trials/
25 23 or 24
26 Functional electric stimulation/
27 functional electric stimulation.mp. [mp=abstract, heading words, title]
28 26 or 27
29 7 and 13 and 17 and 22 and 25
30 29 not 28

APAPsycArticles

(((MAINSUBJECT.EXACT("Afferent Pathways") OR MAINSUBJECT.EXACT("Proprioception") OR MAINSUBJECT.EXACT("Afferent Stimulation") OR (sensory OR sensation OR sensibility OR sens* OR stimulat* OR somatosens* OR propriocept* OR afferent OR mobilization OR mobilisation OR manipulat*)) AND (foot OR leg OR "lower limb" OR "lower extremity") AND (MAINSUBJECT.EXACT("Walking") OR MAINSUBJECT.EXACT("Gait") OR (walk* OR gait OR mobil* OR step OR stance OR ambulat* OR "weight bearing" OR "weight-bearing")) AND ((MAINSUBJECT.EXACT("Cerebrovascular Accidents") OR MAINSUBJECT.EXACT("Traumatic Brain Injury") OR MAINSUBJECT.EXACT("Hemiparesis") AND MAINSUBJECT.EXACT("Hemiplegia")) OR (MAINSUBJECT.EXACT("Cerebrovascular Accidents") OR MAINSUBJECT.EXACT("Traumatic Brain Injury") OR MAINSUBJECT.EXACT("Hemiparesis") AND MAINSUBJECT.EXACT("Hemiplegia")) OR

(MAINSUBJECT.EXACT("Cerebrovascular Accidents") OR MAINSUBJECT.EXACT("Traumatic Brain Injury") OR MAINSUBJECT.EXACT("Hemiparesis") OR MAINSUBJECT.EXACT("Hemiplegia") OR (stroke OR "cereberovascular accident" OR CVA OR "acquired brain injury" OR "head injury" OR TBI OR ABI OR hemiplegia OR hemiparesis OR "upper notor neuron lesion"))) AND (("randomised controlled trial" OR "randomized controlled trial" OR RCT OR "randomised control trial" OR "randomized control trial") OR (MAINSUBJECT.EXACT("Randomized Controlled Trials") OR MAINSUBJECT.EXACT("Randomized Clinical Trials")))) NOT "functional electric stimulation") AND (la.exact("ENG") AND pd(20020101-20220331))
**APAPsycInfo**<br>(((MAINSUBJECT.EXACT("Afferent Pathways") OR MAINSUBJECT.EXACT("Proprioception") OR MAINSUBJECT.EXACT("Afferent Stimulation") OR (sensory OR sensation OR sensibility OR sens* OR stimulat* OR somatosens* OR propriocept* OR afferent OR mobilization OR mobilisation OR manipulat*)) AND (foot OR leg OR "lower limb" OR "lower extremity") AND (MAINSUBJECT.EXACT("Walking") OR MAINSUBJECT.EXACT("Gait") OR (walk* OR gait OR mobil* OR step OR stance OR ambulat* OR "weight bearing" OR "weight-bearing")) AND ((MAINSUBJECT.EXACT("Cerebrovascular Accidents") OR MAINSUBJECT.EXACT("Traumatic Brain Injury") OR MAINSUBJECT.EXACT("Hemiparesis") AND MAINSUBJECT.EXACT("Hemiplegia")) OR (MAINSUBJECT.EXACT("Cerebrovascular Accidents") OR MAINSUBJECT.EXACT("Traumatic Brain Injury") OR MAINSUBJECT.EXACT("Hemiparesis") AND MAINSUBJECT.EXACT("Hemiplegia")) OR (MAINSUBJECT.EXACT("Cerebrovascular Accidents") OR MAINSUBJECT.EXACT("Traumatic Brain Injury") OR MAINSUBJECT.EXACT("Hemiparesis") OR MAINSUBJECT.EXACT("Hemiplegia") OR (stroke OR "cereberovascular accident" OR CVA OR "acquired brain injury" OR "head injury" OR TBI OR ABI OR hemiplegia OR hemiparesis OR "upper notor neuron lesion"))) AND (("randomised controlled trial" OR "randomized controlled trial" OR RCT OR "randomised control trial" OR "randomized control trial") OR (MAINSUBJECT.EXACT("Randomized Controlled Trials") OR MAINSUBJECT.EXACT("Randomized Clinical Trials")))) NOT "functional electric stimulation") AND (la.exact("ENG") AND pd(20020101-20220331))
**CINAHL**<br># Query Limiters/Expanders Last Run Via Results<br>S1 (MH "Stroke") Expanders - Apply equivalent subjects<br>Search modes - Boolean/Phrase Interface - EBSCOhost Research Databases<br>Search Screen - Advanced Search<br>Database - CINAHL Complete<br>S2 (MH "Brain Injuries") Expanders - Apply equivalent subjects<br>Search modes - Boolean/Phrase Interface - EBSCOhost Research Databases<br>Search Screen - Advanced Search<br>Database - CINAHL Complete<br>S3 (MH "Head Injuries") Expanders - Apply equivalent subjects<br>Search modes - Boolean/Phrase Interface - EBSCOhost Research Databases<br>Search Screen - Advanced Search<br>Database - CINAHL Complete<br>S4 (MH "Hemiplegia") Expanders - Apply equivalent subjects<br>Search modes - Boolean/Phrase Interface - EBSCOhost Research Databases<br>Search Screen - Advanced Search<br>Database - CINAHL Complete<br>S5 (MH "Motor Neuron Diseases") Expanders - Apply equivalent subjects<br>Search modes - Boolean/Phrase Interface - EBSCOhost Research Databases<br>Search Screen - Advanced Search<br>Database - CINAHL Complete<br>S6 Stroke OR cerebrovascular accident OR cva OR acquired brain injury OR traumatic brain injury OR head injury OR tbi OR abi OR hemiplegia OR hemiparesis OR upper motor neuron lesion Expanders - Apply equivalent subjects<br>Search modes - Boolean/Phrase Interface - EBSCOhost Research Databases<br>Search Screen - Advanced Search<br>Database - CINAHL Complete<br>S7 S1 OR S2 OR S3 OR S4 OR S5 OR S6 Expanders - Apply equivalent subjects<br>Search modes - Boolean/Phrase Interface - EBSCOhost Research Databases<br>Search Screen - Advanced Search<br>Database - CINAHL Complete<br>S8 (MH "Sensation") Expanders - Apply equivalent subjects<br>Search modes - Boolean/Phrase Interface - EBSCOhost Research Databases

Search Screen - Advanced Search
Database - CINAHL Complete
S9 (MH "Sensory Stimulation") Expanders - Apply equivalent subjects
Search modes - Boolean/Phrase Interface - EBSCOhost Research Databases
Search Screen - Advanced Search
Database - CINAHL Complete
S10 (MH "Somatosensory Disorders") Expanders - Apply equivalent subjects
Search modes - Boolean/Phrase Interface - EBSCOhost Research Databases
Search Screen - Advanced Search
Database - CINAHL Complete
S11 (MH "Proprioception") Expanders - Apply equivalent subjects
Search modes - Boolean/Phrase Interface - EBSCOhost Research Databases
Search Screen - Advanced Search
Database - CINAHL Complete
S12 (MH "Joint Mobilization") Expanders - Apply equivalent subjects
Search modes - Boolean/Phrase Interface - EBSCOhost Research Databases
Search Screen - Advanced Search
Database - CINAHL Complete
S13 sens* OR stimulat* OR somatosens* propriocept* OR afferent OR mobilisation OR mobilization OR mobilize OR mobilise OR manipulat* Expanders - Apply equivalent subjects
Search modes - Boolean/Phrase Interface - EBSCOhost Research Databases
Search Screen - Advanced Search
Database - CINAHL Complete
S14 S8 OR S9 OR S10 OR S11 OR S12 OR S13 Expanders - Apply equivalent subjects
Search modes - Boolean/Phrase Interface - EBSCOhost Research Databases
Search Screen - Advanced Search
Database - CINAHL Complete
S15 (MH "Foot") Expanders - Apply equivalent subjects
Search modes - Boolean/Phrase Interface - EBSCOhost Research Databases
Search Screen - Advanced Search
Database - CINAHL Complete
S16 (MH "Leg") Expanders - Apply equivalent subjects
Search modes - Boolean/Phrase Interface - EBSCOhost Research Databases
Search Screen - Advanced Search
Database - CINAHL Complete
S17 (MH "Lower Extremity") Expanders - Apply equivalent subjects
Search modes - Boolean/Phrase Interface - EBSCOhost Research Databases
Search Screen - Advanced Search
Database - CINAHL Complete
S18 foot OR leg OR lower limb OR lower extremity Expanders - Apply equivalent subjects
Search modes - Boolean/Phrase Interface - EBSCOhost Research Databases
Search Screen - Advanced Search
Database - CINAHL Complete
S19 S15 OR S16 OR S17 OR S18Expanders - Apply equivalent subjects
Search modes - Boolean/Phrase Interface - EBSCOhost Research Databases
Search Screen - Advanced Search
Database - CINAHL Complete
S20 (MH "Walking") Expanders - Apply equivalent subjects
Search modes - Boolean/Phrase Interface - EBSCOhost Research Databases
Search Screen - Advanced Search
Database - CINAHL Complete
S21 (MH "Gait") Expanders - Apply equivalent subjects
Search modes - Boolean/Phrase Interface - EBSCOhost Research Databases
Search Screen - Advanced Search
Database - CINAHL Complete
S22 (MH "Step") Expanders - Apply equivalent subjects
Search modes - Boolean/Phrase Interface - EBSCOhost Research Databases

Search Screen - Advanced Search
Database - CINAHL Complete
S23 (MH "Weight-Bearing") Expanders - Apply equivalent subjects
Search modes - Boolean/Phrase Interface - EBSCOhost Research Databases
Search Screen - Advanced Search
Database - CINAHL Complete
S24 walk* OR gait OR mobil* OR step OR stance OR ambulat* OR weight bearing OR weight-bearing Expanders - Apply equivalent subjects
Search modes - Boolean/Phrase Interface - EBSCOhost Research Databases
Search Screen - Advanced Search
Database - CINAHL Complete
S25 S20 OR S21 OR S22 OR S23 OR S24 Expanders - Apply equivalent subjects
Search modes - Boolean/Phrase Interface - EBSCOhost Research Databases
Search Screen - Advanced Search
Database - CINAHL Complete
S26 (MH "Randomized Controlled Trials") Expanders - Apply equivalent subjects
Search modes - Boolean/Phrase Interface - EBSCOhost Research Databases
Search Screen - Advanced Search
Database - CINAHL Complete
S27 randomised controlled trial* OR randomized controlled trial* OR RCT OR randomized control trial OR randomised controlled trial Expanders - Apply equivalent subjects
Search modes - Boolean/Phrase Interface - EBSCOhost Research Databases
Search Screen - Advanced Search
Database - CINAHL Complete
S28 S26 OR S27 Expanders - Apply equivalent subjects
Search modes - Boolean/Phrase Interface - EBSCOhost Research Databases
Search Screen - Advanced Search
Database - CINAHL Complete
S29 (MH "Electrical Stimulation, Functional") Expanders - Apply equivalent subjects
Search modes - Boolean/Phrase Interface - EBSCOhost Research Databases
Search Screen - Advanced Search
Database - CINAHL Complete
S30 functional electrical stimulation or fes Expanders - Apply equivalent subjects
Search modes - Boolean/Phrase Interface - EBSCOhost Research Databases
Search Screen - Advanced Search
Database - CINAHL Complete
S31 S29 OR S30 Expanders - Apply equivalent subjects
Search modes - Boolean/Phrase Interface - EBSCOhost Research Databases
Search Screen - Advanced Search
Database - CINAHL Complete
S32 S7 AND S14 AND S19 AND S25 AND S28 NOT S31 Expanders - Apply equivalent subjects
Search modes - Boolean/Phrase Interface - EBSCOhost Research Databases
Search Screen - Advanced Search
Database - CINAHL Complete
S33 S7 AND S14 AND S19 AND S25 AND S28 NOT S31 Limiters - Published Date: 20020101-20211231
Expanders - Apply equivalent subjects
Search modes - Boolean/Phrase Interface - EBSCOhost Research Databases
Search Screen - Advanced Search
Database - CINAHL Complete
S34 S7 AND S14 AND S19 AND S25 AND S28 NOT S31 Limiters - Published Date: 20020101-20211231
Expanders - Apply equivalent subjects
Search modes - Boolean/Phrase Interface - EBSCOhost Research Databases
Search Screen - Advanced Search
Database - CINAHL Complete
S35 S7 AND S14 AND S19 AND S25 AND S28 NOT S31 Limiters - Published Date: 20020101-20211231
Expanders - Apply equivalent subjects
Search modes - Boolean/Phrase Interface - EBSCOhost Research Databases

Search Screen - Advanced Search<br>Database - CINAHL Complete<br>S36 S7 AND S14 AND S19 AND S25 AND S28 NOT S31 Limiters - Published Date: 20020101-20220331; English Language; Human<br>Expanders - Apply equivalent subjects<br>Search modes - Boolean/Phrase Interface - EBSCOhost Research Databases<br>Search Screen - Advanced Search<br>Database - CINAHL Complete
**Cochrane Library**<br>#1 (stroke OR "cerebrovascular accident" OR brain injur* OR hemiplegia OR "traumatic brain injury" OR TBI OR "acquired brain injur*" OR ABI):ti,ab,kw AND ("sens* disorder*" OR "electric* stimulation" OR propriocept* or somastosen* or stimulat* or mobil*):ti,ab,kw AND (foot OR leg OR "lower limb*" OR "lower extremit*"):ti,ab,kw AND (walk* OR gait OR "weight-bearing" or "weight bearing" OR mobil* OR step* OR stance OR ambulat*):ti,ab,kw AND ("randomised clinical trial" OR "randomized clinical trial"):ti,ab,kw with (Word variations have been searched)<br><br>#2 #1 NOT "functional electric* stimulation"
**Embase**<br>1 stroke/<br>2 Cerebrovascular accident/<br>3 Brain injuries/<br>4 Head injuries/<br>5 Hemiplegia/<br>6 (stroke or cerebrovascular accident or CVA or acquired brain injury or traumtatic brain injury or head injury or TBI or ABI or hemiplegia or hemiparesis or upper motor neuron lesion).mp. [mp=title, abstract, heading word, drug trade name, original title, device manufacturer, drug manufacturer, device trade name, keyword heading word, floating subheading word, candidate term word]<br>7 1 or 2 or 3 or 4 or 5 or 6<br>8 Sensation disorders/<br>9 Electric stimulation/<br>10 Proprioception/<br>11 Mobilisation/<br>12 (sens* or stimulat* or somatosens* or propriocept* or afferent or mobilisation or manipulat* or sensory or sensation or sensibility).mp. [mp=title, abstract, heading word, drug trade name, original title, device manufacturer, drug manufacturer, device trade name, keyword heading word, floating subheading word, candidate term word]<br>13 8 or 9 or 10 or 11 or 12<br>14 Foot/<br>15 Leg/<br>16 (foot or leg or lower limb or lower extremity).mp. [mp=title, abstract, heading word, drug trade name, original title, device manufacturer, drug manufacturer, device trade name, keyword heading word, floating subheading word, candidate term word]<br>17 14 or 15 or 16<br>18 Walking/<br>19 Gait/<br>20 Weight bearing/<br>21 (walk* or gait or mobil* or step or stance or ambulat* or weight bearing or weight-bearing).mp. [mp=title, abstract, heading word, drug trade name, original title, device manufacturer, drug manufacturer, device trade name, keyword heading word, floating subheading word, candidate term word]<br>22 18 or 19 or 20 or 21<br>23 randomised controlled trial.mp. [mp=title, abstract, heading word, drug trade name, original title, device manufacturer, drug manufacturer, device trade name, keyword heading word, floating subheading word, candidate term word]<br>24 Randomized controlled trials/<br>25 23 or 24<br>26 Functional electric stimulation/

27	functional electric stimulation.mp. [mp=title, abstract, heading word, drug trade name, original title, device manufacturer, drug manufacturer, device trade name, keyword heading word, floating subheading word, candidate term word]
28	26 or 27 2491
29	7 and 13 and 17 and 22 and 25
30	29 not 28
31	limit 30 to (english language and yr="2002 - 2022")
**Emcare**	
1	stroke/
2	Cerebrovascular accident/
3	Brain injuries/
4	Head injuries/
5	Hemiplegia/
6	(stroke or cerebrovascular accident or CVA or acquired brain injury or traumtatic brain injury or head injury or TBI or ABI or hemiplegia or hemiparesis or upper motor neuron lesion).mp. [mp=title, abstract, heading word, drug trade name, original title, device manufacturer, drug manufacturer, device trade name, keyword heading word]
7	1 or 2 or 3 or 4 or 5 or 6
8	Sensation disorders/
9	Electric stimulation/
10	Proprioception/
11	Mobilisation/
12	(sens* or stimulat* or somatosens* or propriocept* or afferent or mobilisation or manipulat* or sensory or sensation or sensibility).mp. [mp=title, abstract, heading word, drug trade name, original title, device manufacturer, drug manufacturer, device trade name, keyword heading word]
13	8 or 9 or 10 or 11 or 12
14	Foot/
15	Leg/
16	(foot or leg or lower limb or lower extremity).mp. [mp=title, abstract, heading word, drug trade name, original title, device manufacturer, drug manufacturer, device trade name, keyword heading word]
17	14 or 15 or 16
18	Walking/
19	Gait/
20	Weight bearing/
21	(walk* or gait or mobil* or step or stance or ambulat* or weight bearing or weight-bearing).mp. [mp=title, abstract, heading word, drug trade name, original title, device manufacturer, drug manufacturer, device trade name, keyword heading word]
22	18 or 19 or 20 or 21
23	randomised controlled trial.mp. [mp=title, abstract, heading word, drug trade name, original title, device manufacturer, drug manufacturer, device trade name, keyword heading word]
24	Randomized controlled trials/
25	23 or 24
26	Functional electric stimulation/
27	functional electric stimulation.mp. [mp=title, abstract, heading word, drug trade name, original title, device manufacturer, drug manufacturer, device trade name, keyword heading word]
28	26 or 27
29	7 and 13 and 17 and 22 and 25
30	29 not 28
**Medline**	
S1	(MH "Stroke")
S2	(MH "Brain Injuries")
S3	(MH "Brain Injuries, Traumatic")
S4	(MH "Head Injuries, Closed") OR (MH "Head Injuries, Penetrating")
S5	(MH "Hemiplegia")
S6	(MH "Motor Neuron Disease")

S7 Stroke OR cerebrovascular accident OR cva OR acquired brain injury OR traumatic brain injury OR head injury OR tbi OR abi OR
S8 S1 OR S2 OR S3 OR S4 OR S5 OR S6 OR S7
S9 (MH "Sensation")
S10 (MH "Physical Stimulation")
S11 (MH "Somatosensory Disorders")
S12 (MH "Proprioception")
S13 (MH "Afferent Pathways")
S14 sens* OR stimulat* OR somatosens*
S15 propriocept* OR afferent OR (mobilisation or mobilization or mobilize or mobilise) OR manipulat*
S16 S9 OR S10 OR S11 OR S12 OR S13 OR S14 OR S15
S17 (MH "Foot")
S18 (MH "Leg")
S19 (MH "Lower Extremity")
S20 foot OR leg OR lower limb OR lower extremity
S21 S17 OR S18 OR S19 OR S20
S22 (MH "Walking")
S23 (MH "Gait")
S24 (MH "Weight-Bearing")
S25 walk* OR gait OR mobil* OR step OR stance OR ambulat* OR weight bearing OR weight-bearing
S26 S22 OR S23 OR S24 OR S25
S27 randomised controlled trial* OR randomized controlled trial* OR RCT OR randomized control trial OR randomised controlled trial
S28 functional electrical stimulation OR FES
S29 S7 AND S16 AND S21 AND S26 AND S27
S30 S29 NOT S28
S31 S29 NOT S28
S32 S29 NOT S28

Limiters - Date of Publication: 20020101-20221231
Expanders - Apply equivalent subjects
English Language

PEDro
Abstract & Title: somatosens* stroke
Body part: Lower leg or knee
Published since: 2002
All terms matched with AND

SPORTDiscus
S1 DE "STROKE"
S2 DE "BRAIN injuries"
S3 DE "HEMIPLEGIA"
S4 stroke OR cerebrovascular accident OR CVA OR acquired brain injur* OR traumatic brain injur* OR head injur* OR TBI OR ABI OR hemiplegia OR hemiparesis OR upper motor neuron lesion
S5 S1 OR S3 OR S4
S6 DE "ELECTRIC stimulation"
S7 DE "PROPRIOCEPTION"
S8 DE "AFFERENT pathways"
S9 DE "MANIPULATION therapy"
S10 sens* OR stimula* OR somatosens* OR propriocept* OR afferent OR mobilisation OR mobilization OR manipulat*
S11 S6 OR S7 OR S8 OR S9 OR S10
S12 DE "FOOT
S13 DE "LEG"
S14 foot OR leg OR lower limb* OR lower extremit*
S15 S12 OR S13 OR S14

S16 DE "WALKING"
S17 DE "GAIT disorders"
S18 DE "WEIGHT-bearing (Orthopedics)"
S19 walk* OR gait OR mobil* OR step OR stance OR ambulat* OR weight bearing OR weight-bearing
S20 S16 OR S17 OR S18 OR S19
S21 randomised controlled trial* OR randomized controlled trial OR RCT OR randomized control trial OR randomised control trial
S22 functional electrical stimulation
S23 S5 AND S11 AND S15 AND S20 AND S21 NOT S22

Web of Science

(((((TS=(stroke OR "cerebrovascular accident" OR "acquired brain injury" OR "traumatic brain injury" OR "head injury" OR TBI OR ABI OR hemiplegia OR hemiparesis OR "upper motor neuron lesion")) AND TS=(sens* OR stimulat* OR somatosens* OR propriocept* OR afferent OR mobilisation OR mobilization OR manipulat* OR sensory OR sensation* OR sensibility)) AND TS=(foot OR leg OR lower extremit* OR lower limb*)) AND TS=(walk* OR gait OR mobil* OR step OR stance OR ambulat* OR "weight bearing" OR weight-bearing)) AND TS=("randomised controlled trial" OR "randomized controlled trial" OR "randomised control trial" OR "randomised control trial")) NOT TS=("functional electrical stimulation")

Database : Web of Science Core Collection

Table S2. Reasons for exclusion of full texts

Study/Report	Reason for exclusion from full text
Alwhaibi et al. (2021)	Visual and auditory stimulation as part of somatosensory training
Bae et al. (2015)	No ethical approval statement in the report
Ertzgaard et al. (2018)	Whole body stimulation, not focal. Another issue was the population – cerebral palsy and stroke mixed.
Hsu et al. (2013)	Active movement from noxious stimulus
In et al. (2021)	No true control group
Kim and Kang, Tae-Woo (2018)	Active movement encouraged
Kwong et al. (2018a)	No true control group
Lau (2013)	Thesis
Lee and Lee Gyuchang (2019)	Active movement encouraged
Lee et al. (2016)	Active movement encouraged
Lou et al. (2019)	No ethical approval statement in the report
Maupas et al. (2017)	Not an RCT, no relevant outcome measure and active movement
Mesci et al. (2009)	NMES (EMG triggered)
Morioka et al. (2009)	No ethical approval statement in the report
Park and Lee (2019)	No ethical approval statement in the report and no true control
Park et al. (2015)	Not an RCT
Seung-Mi Lee et al. (2017)	No true control
Sheffler et al. (2013)	FES
Sheffler et al. (2015)	FES
Sungkarat et al. (2011)	Auditory feedback
Unal et al. (2021)	Treatment combined with exercises (active movement)
van Vliet et al. 2005	Not specifically somatosensory
Xu et al. (2017)	Active movement encouraged
Yavuzer et al. (2006)	NMES with muscle contraction

EMG=Electromyography; FES=Functional electrical stimulation; NMES=Neuromuscular electrical stimulation; RCT=Randomized controlled trial

Table S3. Additional information after screening reference lists and reading full texts of potential articles to include - in preparation for PRISMA flow diagram

Possible articles identified following hand search of reference lists n = 31

Author(s) of report identified to check	Report it was identified from	Reason for not including/including report
Byl et al. (2008)	Paoloni et al. (2010)	Upper limb
Chen et al. (2010)	Ferreira et al. (2018)	Not an RCT
Chen et al. (2011)	Goliwas et al. (2015)	Active movement away from stimulus
Cho et al. (2013)	Suh et al. (2014)	INCLUDE
Choi et al. (2020)	In et al. (2021)	No true control – both groups had taping
Cordo et al. (2009)	Paoloni et al. (2010)	Involved active movement
de Wit et al. (2004)	Ferreira et al. (2018)	Not somatosensory - AFOs
Eckhardt et al. (2011)	Ferreira et al. (2018)	Not an RCT
Esquenazi et al. (2009)	Ferreira et al. (2018)	Not RCT - Retrospective study looking at stroke records
Fatone et al. (2009)	Ferreira et al. (2018)	Not an RCT
Geiger et al. (2001	Goliwas et al. (2015) and Lynch et al. (2007)	Visual feedback
Gok et al. (Alptekin)* et al (2008)	Goliwas et al. (2015)	Involved visual feedback
Gök et al. (2003)	Ferreira et al. (2018)	Not an RCT
Hillier & Dunsford (2006)	Lynch et al. (2007)	Not an RCT – (n=3, case studies)
Julkunen et al. (2005)	Lynch et a.l (2007)	Not an RCT and upper limb
Jung et al. (2017)	In et al. (2021)	INCLUDE
Kluding et al. (2004)	Kluding and Santos (2008)	Not an RCT
Kobayashi et al. (2011)	Ferreira et al. (2018)	Not an RCT
Koseoglu et al. (2017)	In et al. (2021)	
Lee et al. (2013)	Önal et al. (2022)	No true control and involved active movement
Lim et al. (2019)	In et al. (2021)	No true control
Morioka & Yagi (2003)	Lynch et al. (2007)	Exclude – no statement re ethical approval
Ng & Hui Chan (2007)	In et al. (2021)/ Yan and Hui-Chan (2007)	Exclude – no statement re ethical approval
Ng &Hui Chan (2009)	In et al. (2021)/ Suh et al. (2014)	INCLUDE
Park et al. (2014)	Yen et al. (2019)	INCLUDE
Smania et al. (2003)	Lynch et al. (2007)	Upper limb
Steyvers et al. (2003	Paoloni et al. (2010)	Not stroke
Tyson et al. (2013)	Goliwas et al. (2015)/ Suh et al. (2014)	Discussed between AA, PD and SH – cross over trial – excluded because of potential carryover of effects from one group to the other
Yavuzer et al. (2006)	Kluding and Santos (2008)	Exclude – muscle contraction

*Study incorrectly referenced as Alptekin et al. instead of Gok et al. in the reference list of Goliwas et al.

Full texts read and added n= (4) – identified in green font in table S3.

Additional full texts read following screening of reference lists of final four additional included articles:

Author(s) of article identified to check	Report it was identified from	Reason for not including/including report
Chen et al. (2005)	Jung et al. (2017)	No statement of approval
Laddha et al. (2016)	Jung et al. (2017)	Muscle contraction
